# The Effect of Castration on Peripheral Autonomic Neurons Supplying Mammalian Male Genitourinary System

**DOI:** 10.3390/ijms22147632

**Published:** 2021-07-16

**Authors:** Jerzy Kaleczyc, Ewa Lepiarczyk

**Affiliations:** 1 Department of Animal Anatomy, Faculty of Veterinary Medicine, University of Warmia and Mazury in Olsztyn, Oczapowskiego 13, 10-719 Olsztyn, Poland; 2 Department of Human Physiology and Pathophysiology, School of Medicine, University of Warmia and Mazury in Olsztyn, Warszawska 30, 10-082 Olsztyn, Poland

**Keywords:** male genitourinary system, testosterone deprivation, androgen receptor, castration, apoptosis, autonomic innervation

## Abstract

This review paper deals with the influence of androgens (testosterone) on pelvic autonomic pathways in male mammals. The vast majority of the relevant information has been gained in experiments involving castration (testosterone deprivation) performed in male rats, and recently, in male pigs. In both species, testosterone significantly affects the biology of the pathway components, including the pelvic neurons. However, there are great differences between rats and pigs in this respect. The most significant alteration is that testosterone deprivation accomplished a few days after birth results some months later in the excessive loss (approximately 90%) of pelvic and urinary bladder trigone intramural neurons in the male pig, while no changes in the number of pelvic neurons are observed in male rats (rats do not have the intramural ganglia). In the castrated pigs, much greater numbers of pelvic neurons than in the non-castrated animals express CGRP, GAL, VIP (peptides known to have neuroprotective properties), and caspase 3, suggesting that neurons die due to apoptosis triggered by androgen deprivation. In contrast, only some morpho-electrophysiological changes affecting neurons following castration are found in male rats. Certain clinicopathological consequences of testosterone deprivation for the functioning of urogenital organs are also discussed.

## 1. Introduction

Gonadal steroids exert powerful effects on the growth and maintenance of many neurons, and their substantial role in establishing sexually dimorphic neuronal circuits in the mammalian central nervous system has been widely studied and now is quite well understood [1,2,3,4,5]. However, significantly less attention has been paid to steroid action on peripheral neurons, and the ganglia involved in male pelvic nerve pathways are one of the few identified targets of androgens in the peripheral nervous system. Until recently, virtually all the data came from investigations performed in male rats, and the most relevant and comprehensive contributions published in the second half of the 1990s were compiled by Prof. Janet R. Keast and her collaborators. The knowledge, already relatively comprehensive and complete at that time, has been summarized in some excellent reviews [6,7,8,9] and remains valid until today. The above-mentioned studies show that one of the most important issues is identifying which neurons are sensitive to the effects of testosterone. This can be determined using two approaches. The first is to ascertain if neurons express androgen receptor (AR); if so, this allows us to presume that they are directly influenced by testosterone. The second is to examine the effects of androgen deprivation either by surgical or chemical castration. These should reveal the traits typically maintained by androgens.

Recently, a few contributions in the field have been published [10,11,12], which provide some new and interesting findings. First, these studies were performed in male pigs; second, they considered elements of both above-mentioned approaches combining the surgical removal of testes (castration) with immunohistochemical and/or quantitative real-time PCR (qPCR) investigations of AR expression in neurons of the anterior pelvic ganglion (APG) and intramural ganglia of the urinary bladder (UB) trigone (UBT); third, the comparison of the corresponding findings obtained in male rats and pigs reveals substantial differences, which may be of great importance for planning further investigations aimed at obtaining results important for human and veterinary medicine. It should be noted that the pig is obviously an important animal for veterinary medicine and agriculture, but on the other hand, it has become a critically important experimental animal in biomedical research [13].

Therefore, this short review addresses the above issues and especially focuses on the comparative analysis of the influence of castration on the pelvic and UB intramural neurons. It should be borne in mind that while laboratory male rats are castrated for experimental purposes only, surgical or chemical castration are treatments widely used in human and veterinary medicine. Moreover, certain clinicopathological consequences of testosterone deprivation for the functioning of urogenital organs are also discussed.

## 2. Some General Remarks

Bilateral orchidectomy (castration) in male mammals, i.e., removal of male gonads, the testes, is a surgical operation commonly performed in different species due to some important requirements. It is applied in domestic animals, pigs, horses, bovine, cats, and even dogs to achieve specific, depending on the breeding purposes, biological effects. These include mostly either appropriate behavioral (horses, cats, dogs) or dealing with the meat production (pigs, bovine) aspects. Surgical (or pharmacological) castration is also performed in human and veterinary medicine due to therapeutic indications (associated mainly with severe, organic diseases of testes or their neighboring organs, or prostate cancer).

Since, as mentioned, the removal of male gonads causes androgen deprivation, and it has been also applied as a methodological approach in many studies to investigate the significance of androgens for the proper development, morphology, and function of various tissue structures. One of the most interesting aspects of these investigations is dealing with the plasticity of peripheral neurons supplying male urogenital organs associated with surgical castration and/or androgen (testosterone) replacement [9].

The term “neuronal plasticity” has a broad meaning but in general is based on the observation that neurons can change their structure, function, and biochemistry under physiological and pathological conditions [14]. These adaptive (plastic) changes include, among others, both up- and down-regulation of transmitter expression and the induction of new genes in the nerve cells.

As stated earlier, information concerning gonadal steroid actions on peripheral neurons derives almost entirely from previous investigations performed in the male rat major pelvic ganglion (MPG) and, to a lesser extent, the hypogastric ganglion (HG) [8], and recent studies carried out on the male pig APG and intramural ganglia of the UBT [10,11,12].

In the male rat, the MPG consists of either sympathetic adrenergic or parasympathetic cholinergic neurons [15,16] which also express some neuropeptides [17]. The neurons innervate reproductive organs (sexually dimorphic targets) as well as the UB and lower bowel [17,18]. The rat HG is a smaller structure than the MPG (and much more seldom chosen than the MPG for investigations dealing with male rat pelvic ganglia) and contains only adrenergic sympathetic neurons, which project mostly to accessory genital glands [19,20].

**Figure 1:**
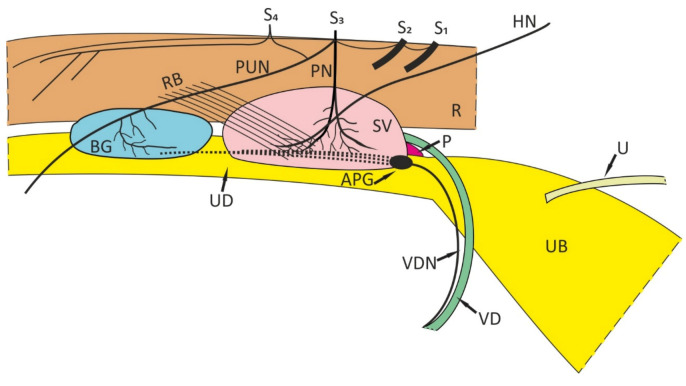
Schematic representation showing the distribution of ganglia in the pelvic plexus of the juvenile male pig. Lateral view. R—rectum, UB—urinary bladder, U—ureter, UD—urogenital duct, VD—vas deferens, SV—seminal vesicle, BG—bulbourethral gland, HN—hypogastric nerve, P—prostate gland, PN—pelvic nerve, PUN—pudendal nerve, RB—rectal branches, VDN—nerve bundle accompanying VD, APG—anterior pelvic ganglion, the dotted lines represent the remaining nerve cell clusters, S1–S4—spinal sacral nerves.

In the male pig, the APG, apparently the largest ganglion of the pelvic plexus, is found between the proximal end of the vas deferens and the caudal part of the seminal vesicle (Figure 1).

The remaining smaller pelvic ganglia are distributed along lateral sides of the pelvic part of the urogenital duct [21]. As with the rat MPG, the APG also comprises adrenergic and cholinergic neurons, which express some neuropeptides. Retrograde tracing studies have revealed that this ganglion supplies the testis [22], vas deferens [21], and UB [23] and some evidence suggests that it also supplies the seminal vesicle and prostate [10].

The number of intramural neurons or ganglia in the UB differs significantly among species; they are numerous in many mammals, including humans and domestic pigs [24,25,26,27,28,29] but are absent in rats and mice [30].

## 3. Androgen-Dependent Peripheral Neurons in Male Rats and Pigs

**Figure 2:**
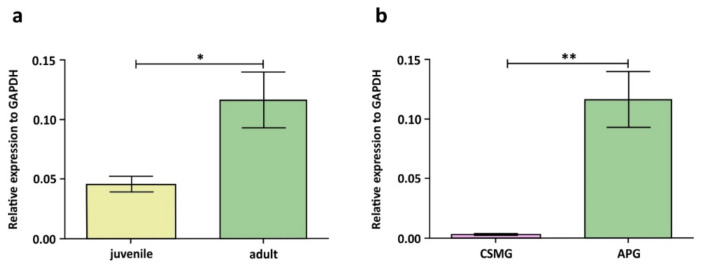
(**a**) Gene expression of androgen receptor (AR) in anterior pelvic ganglion (APG) tissue in 7-day-old (juvenile) and 180-day-old (adult) male pigs measured by quantitative real-time PCR (qPCR). Bars represent means and error bars correspond to SEM. * difference significant at *p* ≤ 0.05. (**b**) Gene expression of AR in APG and celiac-superior mesenteric ganglion (CSMG) tissue in the adult male pigs measured by qPCR. Bars represent means and error bars correspond to SEM. ** difference significant at *p* ≤ 0.01; Figure 2 is adapted from Kaleczyc et al. [31].

Although the mechanisms by which testosterone affects peripheral neurons presumably can be diverse, the presence of AR in them is believed to be a good indicator of their sensitivity to this steroid hormone [8]. It seems that peripheral autonomic and sensory neurons involved in neural pathways to the male urogenital organs are particularly androgen sensitive. This opinion is strongly confirmed with results obtained by Kaleczyc et al. [31], who investigated the expression of AR in neurons of the APG and celiac-superior mesenteric ganglion (CSMG; ganglion not involved in the innervation of pelvic organs) in the male pig with quantitative real-time PCR (qPCR) and immunohistochemistry. qPCR investigations revealed that the level of AR gene expression in the APG tissue was approximately 2.5 times higher in the adult (180-day-old) than in the juvenile (7-day-old) boars (Figure 2a). Furthermore, in both the adult and juvenile animals, it was significantly higher in the APG than in CSMG tissue (as much as 42 and 85 times higher, respectively; see Figure 2b).

**Figure 3:**
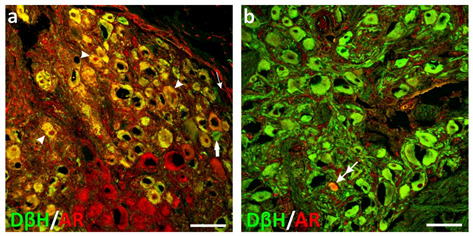
Confocal laser scanning microscope images showing the distribution of AR-positive (AR+; red; Alexa 555 visualization (A555)) and dopamine-β-hydroxylase+ (green; Alexa 488 visualization (A488)) neurons in sections from APG (**a**) and CSMG (**b**) in the adult male pigs. Green and red channels were superimposed (GRDS), double-labeled elements are yellow to orange. In APG, nearly all DβH-positive and many DβH-negative neurons exhibited cytoplasmic and nuclear AR immunoreactivity; arrowheads show some AR-positive neuronal nuclei. Thin and thick arrows show DβH-negative/AR-negative and DβH-positive/AR-negative neurons, respectively. In CSMG, only solitary neurons (double arrow) stained for AR. *Bar* in all images—100 µm; Figure 3 is adapted from Kaleczyc et al. [31].

Immunofluorescence results fully confirmed those obtained with qPCR. In the adult boars, nearly all adrenergic (DβH-positive) and the majority of putative cholinergic neurons in the APG were stained for AR (Figure 3a). In the juvenile animals, about half of the adrenergic and non-adrenergic neurons were AR-positive. In both the adult and juvenile animals, only solitary CSMG neurons stained for AR (Figure 3b). These findings suggest not only that in the male pig, pelvic (but not CSMG!) neurons should be considered as an element of highly testosterone-dependent autonomic circuits involved in the regulation of urogenital function, but also that their sensitization to androgens is a dynamic process, increasing during the prepubertal period. Intriguingly, in the male pig, AR is also expressed in many UBT intramural neurons (32%, 51%, and 81%, in 7-day-, 3-month- and 6-month-old pigs, respectively).

In the male rat, many preganglionic autonomic neurons (in the lumbar and sacral spinal cord) projecting to MPG [32], and many primary sensory neurons in (L6 and S1 dorsal root ganglia) possibly involved in pelvic reflexes [33] express AR immunoreactivity.

Interestingly, in contrast to the findings obtained in male pigs, immunohistochemical studies in the rat major pelvic ganglion (MPG) have failed to identify AR expression in pelvic adrenergic neurons; thus, the mechanism of the androgen action (which, parenthetically, is evident) in this species is not obvious, and one can only presume that testosterone influences these neurons indirectly [8,18]. It has been found that androgen-sensitive are not only pelvic neurons supplying male rat reproductive organs but also some of those (adrenergic) projecting to the UB and rectum [18]. The population of rat MPG cholinergic neurons can be divided into two main, virtually separate, groups distinguished by the presence of vasoactive intestinal peptide (VIP) or neuropeptide Y (NPY) [34]. Interestingly, unlike pelvic adrenergic neurons, which are homogeneously androgen-sensitive (but did not express AR), AR is expressed only by pelvic cholinergic VIP-containing neurons that innervate reproductive organs [18,35]. The cholinergic NPY-positive neurons, which project mainly to the UB and rectum [17], did not contain AR. It is doubtful that similar associations are attributed to APG and UBT intramural cholinergic neurons in the male pig, because earlier investigations have revealed that VIP and NPY are not expressed by separate populations of these nerve cells, respectively, but on the contrary, the majority of cholinergic neurons co-express both peptides (and also nitric oxide synthase and somatostatin) [36]. Therefore, whether AR-positive pelvic cholinergic neurons in the male pig project to reproductive organs only remains to be elucidated with tracing experiments.

It should be noted that although the potency of androgen effects on the nervous system is well accepted, the cellular mechanisms are still poorly understood. As already mentioned, the presence of androgen receptors in neurons is thought to be indicative of their sensitivity to the hormones. However, immunohistochemical studies have failed to identify androgen receptor expression in rat pelvic noradrenergic neurons, which are considered to be uniformly androgen-sensitive [18]. Interestingly, they have been revealed in a population of the cholinergic neurons (thought to be less androgen-sensitive than the noradrenergic neurons) containing vasoactive intestinal polypeptide (VIP) and NOS and mostly projecting to the penis [18,35]. Thus, the correlation between androgen receptor distribution and the neurons that are influenced by castration is imperfect. This suggests that androgens can directly affect the gene expression of some nerve cells but does not prove that this is the mechanism by which they exert their effects. Nevertheless, there are some molecular studies suggesting the possibility of such a correlation [37,38,39]. An in vitro study has revealed that effects caused by testosterone on the structure of many pelvic neurons are mediated indirectly, e.g., by stimulating glial-derived substances; however, they are not mediated by nerve growth factor [40]. Moreover, this study has shown that testosterone influences some of the actions of nerve growth factor, suggesting that there may be complex interactions between steroid signaling and neurotrophic factors in maintaining neuronal structure and function in vivo.

On the other hand, there is also some evidence that estrogens can be synthesized in rat male pelvic ganglia and that the effects of androgens are likely to be at least partly mediated by estrogenic mechanisms [41].

## 4. Effect of Testosterone Deprivation (Castration) on the Number of Peripheral Neurons Supplying Male Urogenital Tract

It has turned out that the extreme consequence of testosterone deprivation (castration) is the death (loss) of neurons. However, the only paper reporting in male rats the loss of peripheral neurons (which probably project not only to testes but also to some other pelvic organs) after surgical castration is the contribution of Melvin et al. [20]. These authors have found that the removal of gonads results in a decrease in the number (approximately 60%) of neurons in the hypogastric ganglion (HG). However, the loss of the neurons was observed (after 12 postoperative weeks) if the surgery was performed on the day of birth (within 12 h). Postnatal castration performed at 10–11 days [19] of age did not cause loss of the neurons after 12 postoperative weeks. Testosterone therapy initiated on the day of the surgery (1st day of life) restored the number of the neurons to normal [20]. However, testosterone replacement was not able to reverse the loss of the nerve cells if the replacement treatment was delayed until day 10. Thus, it seems that testosterone (or estradiol produced from testosterone) prevents neuronal cell death in a critical period immediately after birth. Unfortunately, Melvin et al. [20] did not provide any detailed information about the number of the neurons lost and did not perform any investigations (involving for instance the retrograde tracing method, which is commonly considered to be one of the most advanced and precise approaches in localizing specific neuronal populations supplying any particular organ under study) to find out what population(s) of neurons in the HG were lost in their experiments. The two other papers of Melvin and Hamill [42,43] have confirmed the existence of the “critical perinatal period” for the organization of the rat HG and MPG development. The results obtained by these authors suggest that the adult levels of tyrosine hydroxylase (TH) and choline acetyltransferase (ChAT; key enzymes in catecholamine and acetylcholine synthesis, respectively) activities are organized during prenatal and early postnatal periods and that the biochemical development of pelvic ganglia is highly androgen specific and critically dependent on both the time of exposure and dose of testosterone.

Although, as mentioned before, the literature in the field contains many papers (which will be discussed later) dealing with the consequences of castration (androgen deprivation) and/or testosterone replacement for male rat pelvic neurons, besides the contribution of Melvin et al. [20], none of them provide any information about the neuronal loss, because in case of the studies involving counting of the nerve cells, the removal of gonads was performed after the neonatal critical period.

In contrast to the male rat, in the male pig, castration at age 1 week results in the excessive loss of APG and UB intramural neurons in at least the trigone area [11,12]. The experiment involved 25 male pigs. Ten boars were assigned to routine, surgical castration at 1 week of age following a method commonly applied in veterinary practice. The APGs and UBTs were collected from five intact, 7-day-old animals (IB group), and from five non-castrated (control) and five castrated boars of the corresponding age on the 90th (CoB1 and CaB1 group, respectively) and 180th (CoB2 and CaB2 group, respectively) day after surgery. The number of APG neurons in 3-month-old castrated pigs was decreased (by approximately 32.6%) compared with that found in the control animals of the same age. However, in 6-month-old castrated animals, it was dramatically lower (by approximately 90%) than that determined in the control pigs. In contrast to the consistent appearance of the ganglion found in the remaining animal groups (and also described earlier) [21], it looked completely disintegrated, consisting of small, separated by the fat and connective tissue, clusters of the neurons, comprising from few to several nerve cells. Furthermore, the number of UBT intramural neurons in both castration groups was dramatically lower (by 88% and 87%, respectively) than that in the control group of the same age.

There are several significant indications that the neuronal death observed in the male pigs was due to apoptosis that was most likely triggered by androgen (testosterone) deprivation. Such extensive, apoptotic changes following hormonal deprivation have so far never been reported for peripheral neurons. It should be mentioned that castration performed in male pigs (either juvenile or adult) results in fast, nearly total elimination of testosterone from the blood [44,45,46].

**Figure 4:**
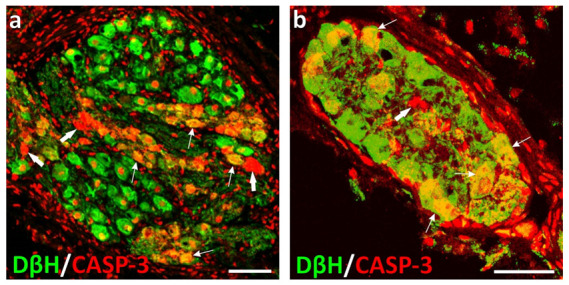
Confocal laser scanning microscope images showing the distribution of CASP-3+ (red; A555) and DβH+ (green; A488) neurons in sections from the APG (**a**) and urinary bladder trigone intramural ganglion (**b**) in the castrated 90-day-old male pigs; GRDS; double-labeled elements are yellow to orange. In the castrated animals, many adrenergic (yellow to orange; some examples are pointed by thin arrows) and some non-adrenergic (cholinergic; red; thick arrows) neurons stained for CASP-3. CASP-3 antibodies unspecifically stained cell nuclei (either neuronal and non-neuronal; information confirmed and provided by the antibody supplier). *Bar* in image a—100 µm; *Bar* in image b—50 µm.

It is well known that apoptosis is coordinated by a family of cysteine proteases known as the caspases. Although many mammalian caspases involved in this process have been identified, CASP-3 is thought to be essential for the accomplishment of the execution phase of apoptosis by cleaving multiple structural and repair proteins [47,48]. In 3- and 6-month-old castrated male pigs, many APG (43% and 24%, respectively; Figure 4a) and UBT intramural (73% and 70%; Figure 4b) neurons displayed immunoreactivity for the cleaved (active) form of this enzyme, and the *casp3* gene was distinctly up-regulated (in non-castrated pigs of the corresponding age, a much smaller number of the neurons expressed CASP-3). Since the excessive loss of the neurons was found in these animals, the conclusion that apoptosis is behind the neuronal death seems to be obvious. Nevertheless, apoptosis was only the most probable way in which the neurons died, but the apparent primary cause was most likely androgen (testosterone) deprivation.

In the male pig, the course of androgen concentrations in blood plasma during fetal and postnatal life is well established [49,50]. In general, the level of testosterone is relatively low throughout the gestational and postnatal periods until puberty. However, before puberty, at least two apparent transient rises in testosterone concentrations have been determined; the one reaches its peak at 35 day of gestation [49], and the second one takes place just after birth, at 3 weeks of age [50]. The animals used in the studies by Kaleczyc et al. [11,12] were castrated at 1 week of age, thus just before the second testosterone surge. It is difficult to speculate whether the time of castration had something to do with the neuronal loss. It should be taken into consideration that in the pig, due to the long gestation period (as compared to that in rats or mice), many developmental processes (such as, for instance, sex differentiation of the central nervous system structures, specifically the hypothalamus, which in small laboratory rodents are completed in postnatal stage), are accomplished before birth [51]. Accordingly, it can be assumed that in the pig, even if it exists, the period essential for the morpho-functional organization of pelvic ganglia with the involvement of gonadal steroids (corresponding to the above-mentioned rat “critical perinatal period”) is completed much earlier than in the rat, already in the prenatal stage, and thus, the time of castration was probably not related to the final findings. This assumption coincides with, for instance, data obtained by Lacorn et al. [45] who have found that castration performed in male pigs at either 1 or 6 weeks of age does not affect the GH-IGF-1 (growth hormone-insulin-like growth factor-1) system, which is the main regulator of anabolic metabolism and growth.

## 5. Effect of Castration on the Chemical Coding of Peripheral Neurons Supplying Male Urogenital Tract

Castration causes changes in morphological [18,19,20], biochemical [19,20,39,42,43,52,53], and electrophysiological [39,54] properties of male rat pelvic neurons, and essentially, all these effects were prevented by testosterone replacement [18,19,20,39,42,43,52,54].

However, interestingly, no alterations in the neurotransmitter phenotype of the pelvic nerve cells investigated with immunohistochemistry have been determined [18], and thus, no corresponding information obtained with the qPCR technique is available. Consequently, the studies by Kaleczyc et al. [11,12] are the first to report remarkable plastic changes in neurotransmitter profiles of male pelvic neurons and UB intramural neurons following castration revealed with immunohistochemistry and qPCR method.

The porcine APG has been immunohistochemically well characterized in intact both juvenile and adult male pigs [21,23,46]. In the 3- and 6-month-old castrated animals, the percentages of adrenergic and cholinergic neurons were similar (33% and 35%, and 67% and 65%, respectively) but differed significantly from those found in the control boars of the same age (67% and 67%, and 33% and 33%, respectively). The percentage ratios between the two populations of the neurons were quite opposite in the castrated animals, which suggests that mostly adrenergic neurons were affected by apoptosis (which, in turn, corresponds well to the results on AR expression in APG neurons; see the earlier comment). In addition to the variations in CASP-3 expression mentioned earlier, the most remarkable other differences observed in the castrated animals were those that pertained to the expression of calcitonin gene-related peptide (CGRP), galanin (GAL), and VIP, which are all peptides known to have, besides the involvement in neurotransmission, neuroprotective and antiapoptotic properties [55,56,57,58,59,60].

In 3-month-old castrated pigs, many cholinergic neurons were stained for GAL, CGRP (these peptides are faintly expressed by APG cholinergic neurons in the control animals), and VIP. GAL was also expressed by much more adrenergic neurons than in the non-castrated pigs. Moreover, many adrenergic neurons displayed immunoreactivity to VIP, which is normally not expressed in this nerve cell population. The qPCR results largely corresponded to the immunofluorescence findings. In the castrated animals, the expression levels of genes for substances that were, compared to the control animals, immunohistochemically detected in greater number of neurons, were distinctly up-regulated. Expectedly, this observation pertains especially to CASP-3 and CGRP and provides further evidence that many APG (mostly adrenergic) neurons were undergoing apoptosis, while some others tried to survive and expressed neuroprotective peptides, CGRP, GAL, or VIP. Along with the immunohistochemical ascertainment, *DβH* and *VAChT* gene expression levels were down- or up-regulated, respectively; however, the differences were statistically insignificant.

The APG in 6-month-old castrated pigs appeared to be practically completely disintegrated. The vast majority of the neurons were lost and replaced with fat and connective tissue. CGRP was expressed by many cholinergic neurons. Immunoreactivity to GAL or VIP was found in many adrenergic and cholinergic neuronal somata. The qPCR results also largely corroborated the immunofluorescence findings. In the castrated pigs, genes for GAL, CGRP, and VIP were again found to be distinctly up-regulated. This would suggest that for at least some surviving neurons, the trouble has not yet ended. They tried to survive by expressing neuroprotective substances, and their altered metabolism was reflected by the rise in the expression levels of genes for DβH and VAChT.

Urinary bladder intramural neurons are thought to represent an extension of the pelvic plexus [24,28]. This seems to be justified considering, for instance, the similarities in the neurochemical properties of ganglia found in these two domains. In both cases, they are “mixed” autonomic ganglia consisting of either sympathetic adrenergic or parasympathetic cholinergic neurons. Recent investigations of Kaleczyc et al. [12] and earlier findings [61], which have revealed that the vast majority of UBT intramural neurons in male pigs are either adrenergic or cholinergic in nature, seem to further confirm this concept. No wonder then that changes in percentages of adrenergic and cholinergic neurons observed after castration [12] were similar to those found in the APG. In the 3- and 6-month-old castrated male pigs, the percentages of adrenergic and cholinergic neurons were comparable (41% and 39%, and 58% and 58%, respectively) but they differed significantly from those determined in the non-castrated animals of the same age (68% and 68%, and 31% and 31%, respectively). Again, the percentage ratios between the two populations of the neurons were quite the opposite in the castrated animals, which suggests that apoptosis affected mostly adrenergic neurons. The authors of this contribution had to reduce the number of substances investigated because of the very small number of neurons to be analysed in the castrated animals. Therefore, no information is available on the expression of neuropeptides (as mentioned earlier, the expression of AR and CASP-3 was also investigated), which, for instance, are known to have neuroprotective and antiapoptotic properties (see earlier comment on the expression of such substances in the APG).

It should also be mentioned that in the male pigs, castration resulted in a remarkable loss of intraganglionic, especially cholinergic (APG, UBT intramural ganglia) and CGRP/SP-positive (APG) nerve fibers (presumably preganglionic and collaterals of spinal ganglia neurons, respectively) [62], which suggests that the disintegration also affected preganglionic neurons and thus could have broader functional implications. In this context, it should be recalled that in rats, many preganglionic autonomic and primary sensory neurons possibly involved in pelvic reflexes express immunoreactivity to AR, thus constituting potential targets for circulating testosterone [32,33].

## 6. Conclusions

It appears that castration performed in males of larger mammalian species may be much more harmful than previously believed. It can result in the excessive loss of pelvic neurons, which presumably supply urogenital organs including the UB and urethra, and UB intramural neurons in at least the trigone area. Therefore, these organs become deprived of an important part of their innervation. Since these changes are almost certainly a consequence of gonadal steroid deprivation, it is tempting to assume that they may occur following not only surgical but also any form of hormonal castration, and not only in males but also in females. Thus, it can be further speculated that in larger mammalian species (including humans) gonadectomy, or more generally, reproductive hormone disorders can lead to the specific partial denervation of lower urinary tract organ tissues, which, in turn, may cause problems in their proper functioning. It should be emphasized that the literature dealing with morphofunctional abnormalities concerning especially the UB and urethra observed following gonadectomy (steroid deprivation) or associated with the hormonal disorders is relatively immense [63,64,65,66,67,68,69,70].

Furthermore, the potential neuronal loss-derived unfavorable consequences should be taken into account while applying certain forms of treatments, such as those employed in prostate cancer, involving the elimination of circulating testosterone. It should also be noted that UBT intramural neurons are probably involved in the neural control of the urethral sphincter [71,72]; thus, their loss can have a negative impact on urinary continence status.

Obviously, the above-mentioned assumptions require comprehensive research validation, and the subsequent studies should seek to answer the following questions:

- Would the neuronal loss found in male pigs castrated a few days after birth be observed also in the castrated adult animals?
- Which structures are innervated by the porcine male apoptotic neurons?
- Is castration followed by significant loss of nerve fibers in the organs of the porcine male urogenital system (in either juvenile or adult individuals)?
- In adult individuals of both sexes of other species (including humans), can any significant loss of pelvic or UB intramural neurons be observed after castration (female and male cats, male horses) or following/during natural (menopausal women) or therapeutic (prostate cancer patients) sex steroid deprivation? The same question applies to nerve fibers, especially those supplying the pelvic organs.

Accordingly, the possible results would (a) significantly expand the knowledge on the peripheral neuroendocrine relationships (as mentioned, the corresponding information on the central mechanisms is much more extensive), (b) provide information that can contribute to, or even revise, the current view on causes of disorders in the functioning of the lower urinary tract following gonadectomy (and those associated with severe hormonal disorders) not only in animals but also in humans, and (c) contribute to the veterinary knowledge about the possible consequences of both surgical or pharmacological gonadectomy commonly performed in domestic animals due to various, other than experimental, requirements.
